# Supplementary material for: Synthesis, Structure, and Spectral-Luminescent Properties of Peripherally Fluorinated Mg(II) and Zn(II) Octaphenyltetraazaporphyrins
Source: Molecules. 2022 Dec 6;27(23):8619. doi: 10.3390/molecules27238619 (PMC9739735; doi:10.3390/molecules27238619)
Supplement: Supplementary file 1 [file molecules-27-08619-s001.zip › molecules-2011990-SI.pdf]

Supplementary Information

of the article «Synthesis, Structure, and Spectral-Luminescent Properties of Peripherally Fluorinated Mg(II) and Zn(II) Octaphenyltetraazaporphyrins»

Alexey Rusanov[1,2], Natalya Chizhova[1] and Nugzar Mamardashvili[1*]

[1]G.A. Krestov Institute of Solution Chemistry of the Russian Academy of Sciences, Russia, 153045, Ivanovo,
Akademicheskaya St. 1, [2]Ivanovo State University of Chemistry and Technology Russia, 153460, Ivanovo,
Sheremetevsky Pr. 7
* Correspondence: nugzarstrasburg@mail.ru; Tel.: +7-9038893456

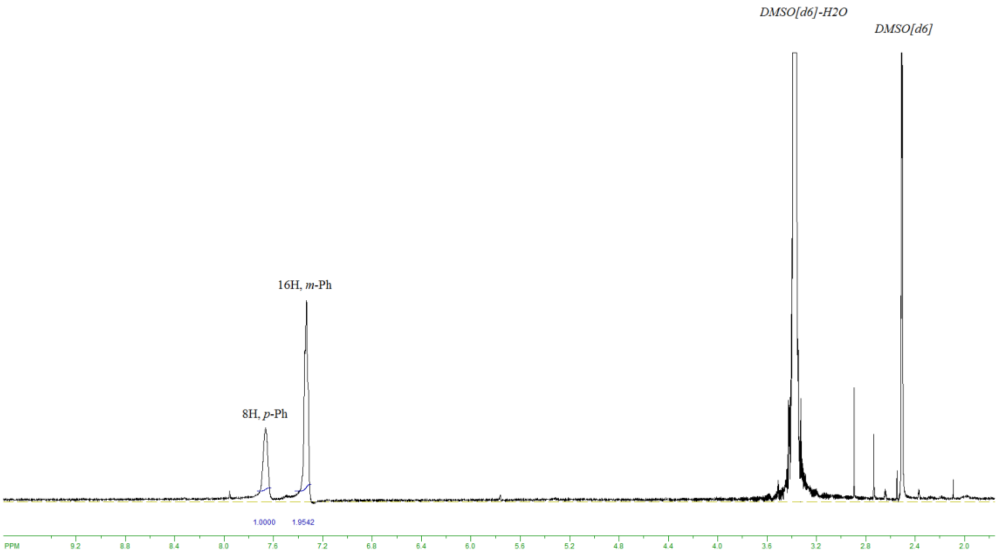

Figure S1. ^{1}H NMR spectrum of the Mg(II)-octa-(2,6-difluorophenyl)tetraazaporphyrin in d_6 DMSO.

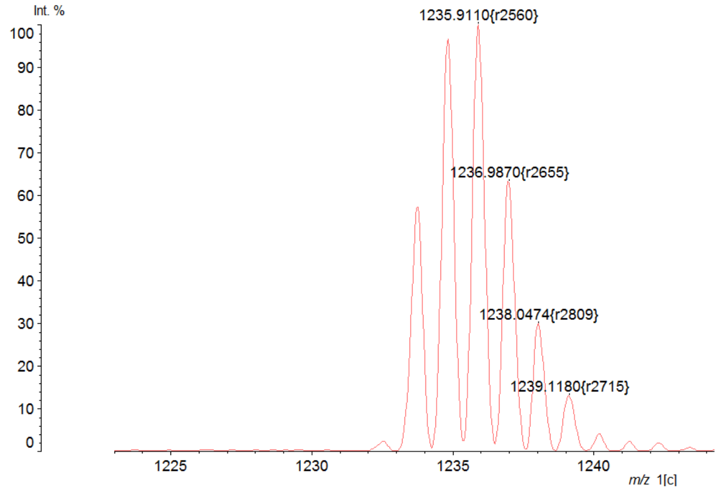

Figure S2. The isotopic distribution of the molecular ion peak of Mg(II)-octa-(2,6-difluorophenyl)tetraazaporphyrin.

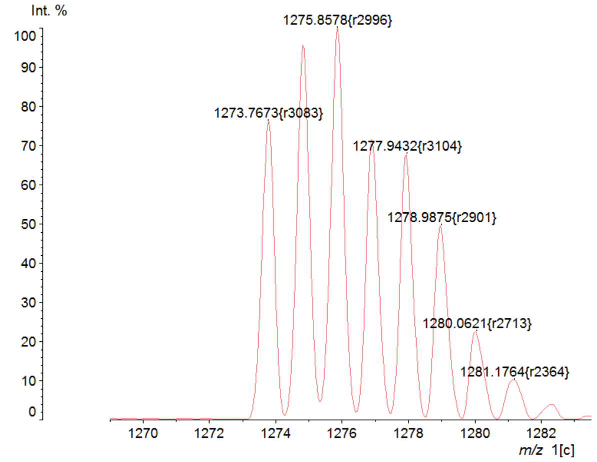

Figure S3. The isotopic distribution of the molecular ion peak of Zn(II)-octa-(2,6-difluorophenyl)tetraazaporphyrin.

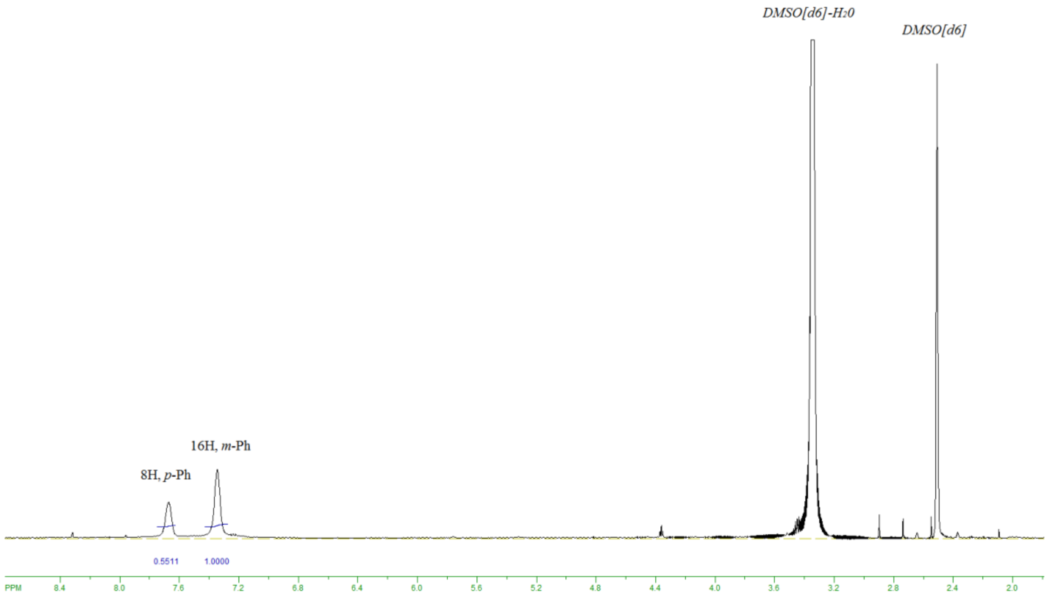

Figure S4. ^{1}H NMR spectrum of the Zn(II)-octa-(2,6-difluorophenyl)tetraazaporphyrin in d_6 DMSO.

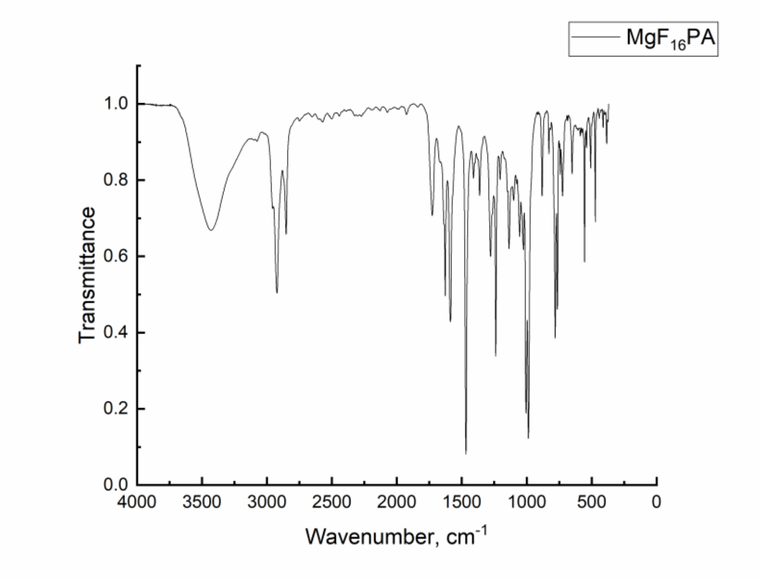

Figure S5. IR spectrum of the Mg(II)-octa-(2,6-difluorophenyl)tetraazaporphyrin in KBr tablets.

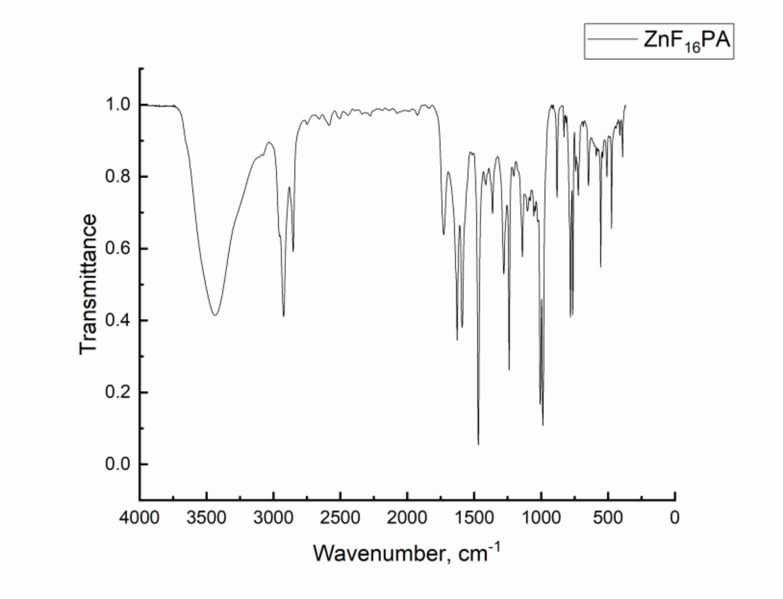

Figure S6. IR spectrum of the Zn(II)-octa-(2,6-difluorophenyl)tetraazaporphyrin in KBr tablets.

Table S1. The values from visualization of the planarity measure and equations of them.

Atom	Values for $MgF_{16}PA$ (**1**)	Values for $ZnF_{16}PA$ (**2**)	Equation **2**/**1**
Metal-ion	0.000	0.140	0.140 (div. by 0)
N	0.005	0.012	2.4
	0.005	0.015	3
	0.005	0.015	3
	0.005	0.013	2.6
Nα	0.000	0.014	0.014 (div. by 0)
	0.000	0.005	0.005(div. by 0)

	0.000<br>0.000	0.007<br>0.005	0.007(div. by 0)<br>0.005(div. by 0)
C	0.026; 0.008<br>0.008; 0.026<br>0.026; 0.008<br>0.008; 0.026	0.014; 0.026<br>0.029; 0.005<br>0.005; 0.029<br>0.026; 0.013	0.5; 3.25<br>3.6; 0.2<br>0.2; 3.6<br>3.2; 0.5

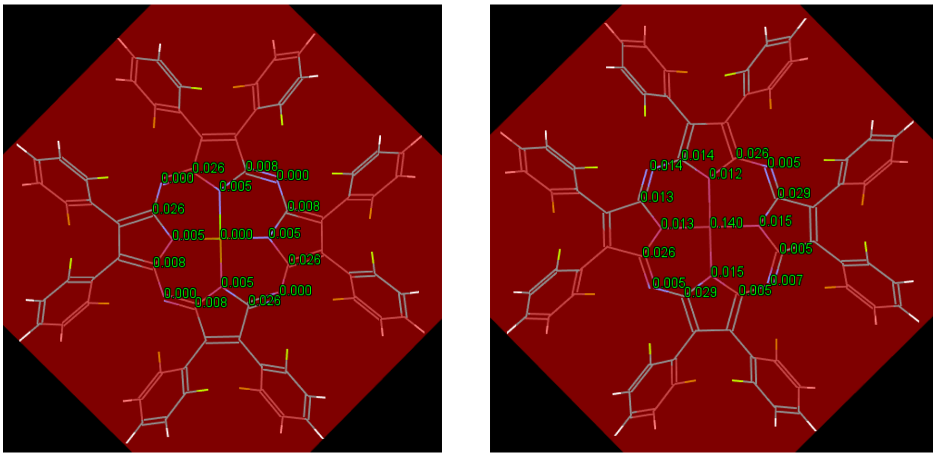

$MgF_{16}PA$ $ZnF_{16}PA$

Figure S7. Visualization of the planarity measure of the Mg(II)-octa-(2,6-difluorophenyl)tetraazaporphyrin and Zn(II)-octa-(2,6-difluorophenyl)tetraazaporphyrin.

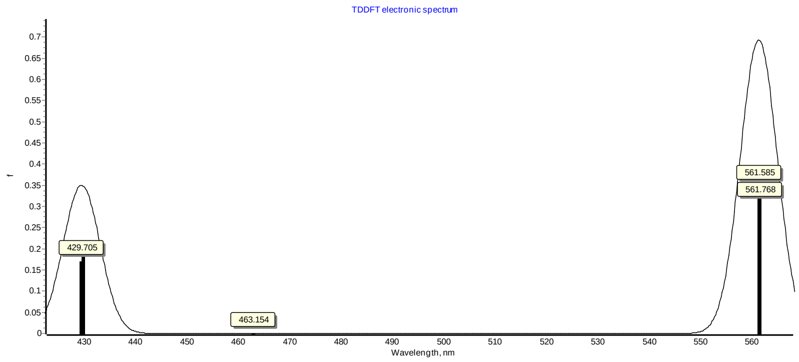

Figure S8. Predicted UV-Vis spectrum of the Mg(II)-octa-(2,6-difluorophenyl)tetraazaporphyrin with nstate=10 and root=1 parameters.

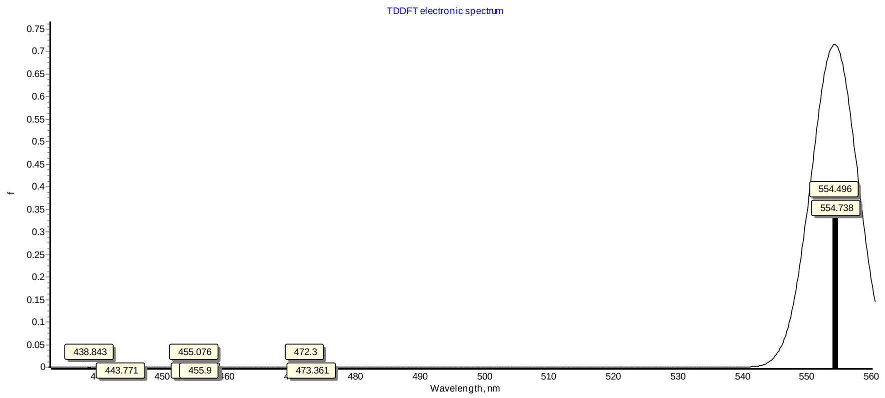

Figure S9. Predicted UV-Vis spectrum of the Zn(II)-octa-(2,6-difluorophenyl)tetraazaporphyrin with nstate=10 и root=1 parameters.

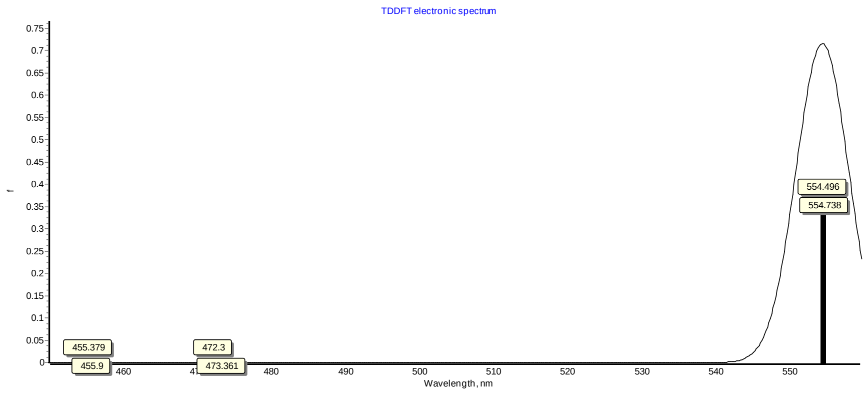

Figure S10. Predicted UV-Vis spectrum of the Zn(II)-octa-(2,6-difluorophenyl)tetraazaporphyrin with nstate=6 и root=20 parameters.

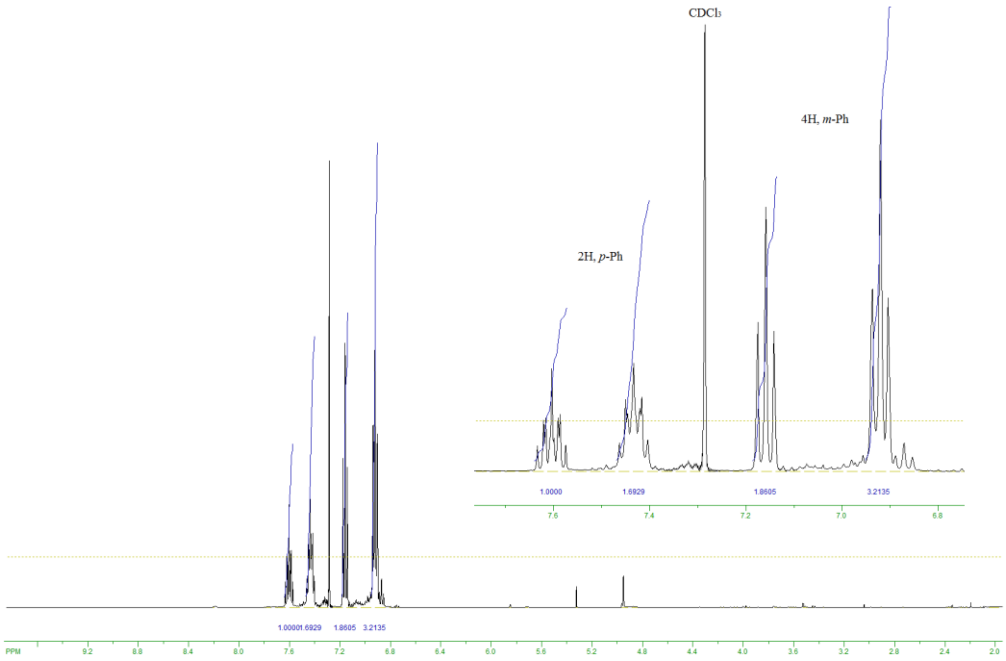

Figure S11. ^{1}H NMR spectrum of the di-(2,6-difluorophenyl)maleindinitrile in $CDCl_3$.

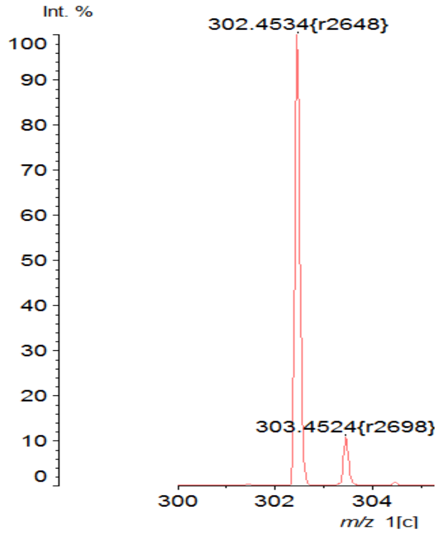

Figure S12. Mass-spectrum of the di-(2,6-difluorophenyl)maleindinitrile.
